# Real-Time Detection of Freezing Motions in Parkinson's Patients for Adaptive Gait Phase Synchronous Cueing

**DOI:** 10.3389/fneur.2021.720516

**Published:** 2021-12-06

**Authors:** Ardit Dvorani, Vivian Waldheim, Magdalena C. E. Jochner, Christina Salchow-Hömmen, Jonas Meyer-Ohle, Andrea A. Kühn, Nikolaus Wenger, Thomas Schauer

**Affiliations:** ^1^Control Systems Group, Technische Universität Berlin, Berlin, Germany; ^2^SensorStim Neurotechnology GmbH, Berlin, Germany; ^3^Department of Neurology, Charité–Universitätsmedizin Berlin, Berlin, Germany

**Keywords:** Parkinson, freezing of gait, wearables, inertial measurement unit, machine learning, neurorehabilitation, on-demand cueing, automation

## Abstract

Parkinson's disease is the second most common neurodegenerative disease worldwide reducing cognitive and motoric abilities of affected persons. Freezing of Gait (FoG) is one of the severe symptoms that is observed in the late stages of the disease and considerably impairs the mobility of the person and raises the risk of falls. Due to the pathology and heterogeneity of the Parkinsonian gait cycle, especially in the case of freezing episodes, the detection of the gait phases with wearables is challenging in Parkinson's disease. This is addressed by introducing a state-automaton-based algorithm for the detection of the foot's motion phases using a shoe-placed inertial sensor. Machine-learning-based methods are investigated to classify the actual motion phase as normal or FoG-affected and to predict the outcome for the next motion phase. For this purpose, spatio-temporal gait and signal parameters are determined from the segmented movement phases. In this context, inertial sensor fusion is applied to the foot's 3D acceleration and rate of turn. Support Vector Machine (SVM) and AdaBoost classifiers have been trained on the data of 16 Parkinson's patients who had shown FoG episodes during a clinical freezing-provoking assessment course. Two clinical experts rated the video-recorded trials and marked episodes with festination, shank trembling, shuffling, or akinesia. Motion phases inside such episodes were labeled as FoG-affected. The classifiers were evaluated using leave-one-patient-out cross-validation. No statistically significant differences could be observed between the different classifiers for FoG detection (*p*>0.05). An SVM model with 10 features of the actual and two preceding motion phases achieved the highest average performance with 88.5 ± 5.8% sensitivity, 83.3 ± 17.1% specificity, and 92.8 ± 5.9% Area Under the Curve (AUC). The performance of predicting the behavior of the next motion phase was significantly lower compared to the detection classifiers. No statistically significant differences were found between all prediction models. An SVM-predictor with features from the two preceding motion phases had with 81.6 ± 7.7% sensitivity, 70.3 ± 18.4% specificity, and 82.8 ± 7.1% AUC the best average performance. The developed methods enable motion-phase-based FoG detection and prediction and can be utilized for closed-loop systems that provide on-demand gait-phase-synchronous cueing to mitigate FoG symptoms and to prevent complete motoric blockades.

## 1. Introduction

Parkinson's disease (PD) is the second most common age-related neurodegenerative disease and the most common movement disorder (1). It is characterized by four cardinal and motor disabling symptoms, bradykinesia, rigidity, tremor, and postural instability. Deuschl et al. (2) reported that the prevalence of PD worldwide is 8.5 million in 2017. The global incidence ranges between 0.85 and 1.2 million. PD represents a major challenge for society, manifested by an increasing strain on the health care structures and the economy (3, 4). The progression of the disease affects the patients heavily. The quality of life and participation in social life are restricted due to the impairment in mobility. No cure is available at the moment for PD. The most common treatment is dopaminergic medication. The effects of dopaminergic medications diminish over time and become less beneficial with the development of the disease. Higher doses of dopaminergic medication can result in induced dyskinesia (involuntary movements), which can limit the dose of the medication (5). Gait and posture impairments are often resistant to the pharmacological treatment, and worsen as the disease progresses.

One of the most disabling symptoms of PD is Freezing of Gait (FoG), which typically arises in the late stages of the disease. FoG appears in episodes during which a severe impairment of mobility or a motoric blockade occurs despite the intention to move. FoG episodes are described by patients as “as if the feet were glued to the floor” (6). Ge et al. (7) reported that the average prevalence of FoG in PD is 39.9% and that with disease progression and duration an increasing prevalence is associated. The highest prevalence of FoG at 70.8% was observed in patients who were affected for 10 or more than 10 years by PD. FoG episodes are brief episodes in the range of only a few seconds. Longer episodes that extend an interval of 30 s are considered rare (8). Due to FoG, patients are exposed to a higher risk of falls, which can lead to physical injuries, fractures, disabilities, and even death (9). The causes of FoG are still debated and remain to be revealed (10), although some scenarios are reported to trigger FoG episodes, such as gait initiation, turning, arriving at a destination, walking through narrow spaces such as doors, and frames, and avoiding obstacles. These scenarios are used in various clinical trials to provoke FoG episodes such as in the test proposed by Ziegler et al. (11). Different subtypes of FoG symptoms can be identified and categorized into akinesia (lack of movement), festination (characterized by small, high frequent steps), shank trembling (inability to move followed by tremor in the affected lower limb), and shuffling (inadequate lifting of the feet off the ground during steps). Shuffling and festination often occur together. Shank trembling, festination, and shuffling are the most common types. Akinesia is rarely observed and its presence is mostly observed in severe cases of PD (8).

A non-invasive treatment for gait disorders and FoG is the application of external stimuli also referred to as cueing. The use of external stimuli in different modalities (acoustic, visual, somatosensory, etc.) can counter FoG symptoms and re-initiate movement as well as improve gait parameters such as step length or cadence [see, e.g., (12–14)]. Cueing can be deployed by using fixed cueing patterns or synchronized to gait events/phases. In some publications, e.g., in Mancini et al. (12), this is named “open loop” and “closed loop,” respectively. Another important classification is whether cueing is applied consistently, i.e., regardless of the presence of FoG symptoms, or adaptively (on-demand), when FoG symptoms occur or have been predicted. Also here, the terms “open loop” and “closed loop” are sometimes used to distinguish these two modes (14). The benefits of consistent cueing may be limited, since cues are provided irrespective of how well a person follows them. Adaptive cueing provides feedback in real-time to the user about performance which may help to improve movement patterns (14). In addition, for usage in daily life, consistent cueing might be distracting and a habituation might mitigate positive effects on the gait.

Recent clinical studies indicate that consistent gait-phase-synchronous electrical or tactile stimulation can successfully improve spatio-temporal gait parameters and consequently reduce FoG systems in PD (12, 15–18). Such closed-loop systems require sensors, like simple foot switches or inertial measurement units, to detect gait events and phases. The stimulation intensity can be at a somatosensory level or at a higher level causing functional muscle contractions. For the latter, drop foot stimulation, i.e. the induction of foot lift by electrical stimulation of the N. peroneus during the swing phase of gait, is one example.

None of the reported studies on gait-phase-synchronous stimulation applied on-demand cueing. Taylor et al. (18) used a commercially available drop foot stimulator which is usually prescribed as a technical aid to stroke patients for permanent use. For daily use in PD, an adaptive form of cueing is highly recommended as gait patterns and deficits fluctuate over the day depending on the current effect of medication and on/off states. The adaptive initiation of cueing by the early detection or even prediction of festination, shuffling, shank trembling might help to reduce or even stop these symptoms and to prevent akinesia.

The on-demand approach requires real-time capable sensor systems and methods for FoG detection or even prediction. In the last years, there have been many reports on developed methods, ranging from simple threshold-based methods to deep learning models [see (19) for an overview]. To motivate our own research, we will shortly review methods solely based on inertial sensors placed at the hip or lower limbs as such a setup enables also a real-time detection of gait events and phases for closed-loop cueing. Inertial sensors are becoming increasingly widespread as they capture movement in 3D, are wireless, low cost, lightweight, and easy to wear on the body. A review of methods for gait phase detection can be found in (20).

Most existing FoG detection algorithms determine signal features in the time and frequency domain from a sliding time window. Moore et al. (21) calculated a parameter called Freezing Index (FI) by dividing the frequency spectrum of the vertical leg acceleration of a shank-worn sensor in a normal locomotor band (0–3 Hz) and a freeze band (3–8 Hz). The FI was calculated as the ratio of spectral power of the two bands in a window of 6 s. The threshold for FI was set individually for each patient. Bächlin et al. (22) extended this method by another threshold parameter to detect standing and walking. They also reduced the window size to 4 s to lower the latency. They reported FoG detection achieved 73% sensitivity and 81% specificity. To improve the FoG detection in window-based approaches many researchers tried using various machine learning techniques. Mazilu et al. (23) extracted various features from the inertial data at different body positions in order to find the best position for detecting FoG episodes. They achieved 66% sensitivity and 95% specificity using patient-independent Random Forest and AdaBoost classifiers. In Naghavi et al. (24), two accelerometers were placed at the ankles and several patient-independent classifiers were trained on the data of seven patients. The classifiers used frequency and time-domain features from a sliding windows of 2 s. A K-Nearest-Neighbors (KNN) model showed the best sensitivity of 90% with a specificity of 82%. Reches et al. (25) trained a Support Vector Machine (SVM) classifier with various time and frequency-domain features extracted from inertial sensors at the ankle and lower back. The reported results were 80% sensitivity and 82.5% specificity. Camps et al. (26) used a Convolutional Neural Network (CNN) model trained on the inertial data of the left waist. The model achieved an accuracy of 89%. Sigcha et al. (27) used the same data set as Camps et al. (26) to train a combined CNN and Recurrent Neural Network (RNN) model. They reported an accuracy of 85%. All window-based approaches suffer from a relatively high latency caused by the window size. This fact limits the use in closed-loop cueing systems where gait phases are usually much shorter than the latency of FoG detection. Therefore, it is inevitable that some gait phases affected by FoG will remain without the supporting stimuli. Furthermore, it is challenging to implement CNNs on a standard low-power microcontroller with limited memory in a wearable device. In addition, deep learning approaches, like CNN, require abundant data sets that are difficult to obtain from clinical trials only. Data collected during activities of daily living are more suitable but often suffer from missing labels.

The following two approaches do not use sliding windows for feature extraction. Borzì et al. (28) equipped patients with two inertial sensors placed on each shin and performed a step-to-step segmentation of the angular velocity signals and subsequent feature extraction in both time and frequency domain. The presented FoG detection algorithm has a reduced latency. As for pre-FoG detection, the implemented classification algorithm achieved 84.1% (85.5%) sensitivity, 85.9% (86.3%) specificity, and 85.5% (86.1%) accuracy in patients on (off) therapy. Suppa et al. (29) used two inertial sensors at both shins and an algorithm based on a time-domain analysis of the fused sensor signals. For a patient-individual manual tuning of the model thresholds, a sensitivity of 93.41% and specificity of 98.51% have been reported.

Only a few studies used spatio-temporal gait parameters from inertial sensor fusion for real-time FoG detection. Azevedo Coste et al. (30) proposed a new real-valued parameter called FoG Criterion based on the estimated step length and observed cadence. FoG is then detected based on a simple threshold condition. In Dvorani et al. (31), also a threshold-based approach is introduced with a real-valued parameter, named GaitScore, to judge a motion phase of the foot as normal or FoG-affected. The GaitScore uses the observed extrema of the pitch angle during the foot's motion phases. In Ginis et al. (32), estimated spatio-temporal parameters have been employed to provide an on-demand auditory cueing or feedback to PD patients when the parameter left predefined healthy ranges. Djurić-Jovičić et al. (33) developed a rule-based system with estimated spatio-temporal gait parameters as inputs derived from two inertial sensors placed laterally along the shank segment of each leg linked with an automatic gait segmentation. Subtypes of FoG have been detected with sensitivities above 78% and specificities above 94%. By now, the tuning of the thresholds/ranges of the listed approaches with spatio-temporal gait parameters is cumbersome and a patient individual tuning is recommended for optimal results. The derived scores in Dvorani et al. (31) and Azevedo Coste et al. (30) combine several estimated parameters by using gait-expert-motivated equations. The optimality of such equations is not guaranteed. The latency of approaches with spatio-temporal gait parameters is not fixed and depends on the gait phase duration. This should be in general shorter than the latency of window-based approaches.

In this contribution, we investigate for the first time the use of spatio-temporal gait parameters for machine learning to classify a completed foot motion phase into normal or FoG-affected. We believe that such a procedure will exploit the given features in the best possible manner for FoG-detection and that the resulting classifier will be useful for on-demand gait-phase-synchronous cueing. The used spatio-temporal gait features are calculated from the actual motion phase after its completion and from optional older motion phases. In addition, we aim to predict whether the next motion phase will show FoG symptoms by using features of previous phases. The gait phase detection introduced by Dvorani et al. (31) will be deployed as the basis to segment the motion phases. Finally, the derived classifiers for FoG detection and prediction will be discussed with respect to their implementation effort on wearable closed-loop stimulation systems.

In the following Chapter 2, the underlying gait-phase-detection algorithm and the machine-learning-based detection/prediction of FoG motion phases are described. After that, the data set and the evaluation methods are presented. The results are reported in Chapter 3 followed by a discussion and conclusions.

## 2. Methods

### 2.1. Gait Phase Detection

**Figure 1:**
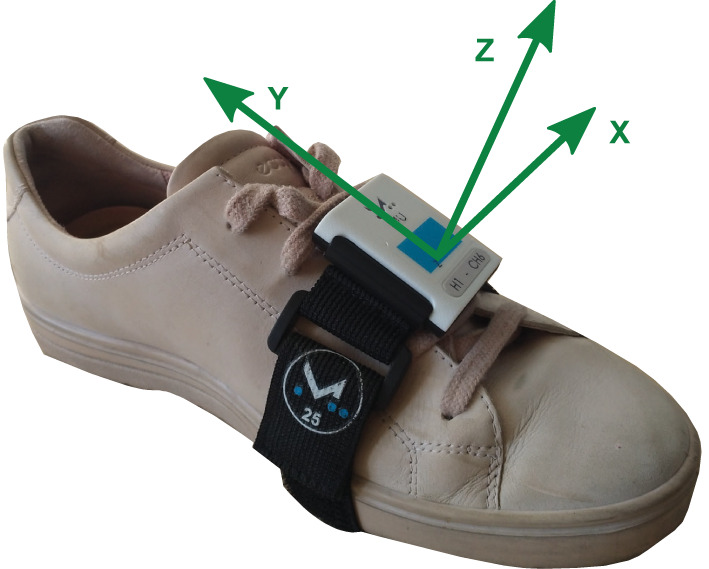
Inertial sensor position and orientation.

**Figure 2:**
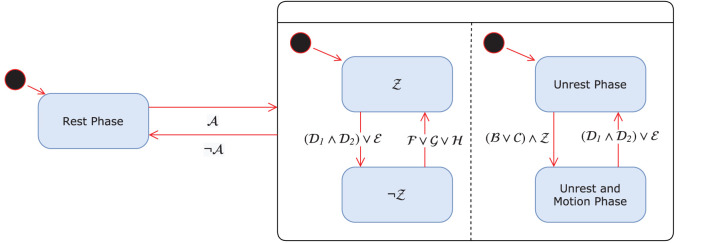
GPD state machine.

The gait-phase-detection algorithm (GPD), initially proposed in Dvorani et al. (31), detects the gait phases based on the kinematic data of the foot recorded with an inertial sensor positioned on the foot's instep. The orientation of the sensor is fixed with the z-axis perpendicular to the foot pointing upwards and the y-axis pointing in the negative walking direction. The position and orientation of the inertial sensor are shown in Figure 1. Inputs to the algorithm are the linear acceleration vector *a*(*t*) ∈ **R**^3^ and angular velocity vector ω(*t*) ∈ **R**^3^ of the foot at a sample rate of 200 Hz. The algorithm detects three phases of the Parkinsonian gait, namely rest phase, during which no foot activities are recorded, unrest, and motion phase. The latter represents a sub-phase of the unrest phase and indicates an effective displacement or orientation change. During the unrest phase, foot activities are detected. Assuming a healthy gait cycle, the motion phase would correspond to the swing phase, with the beginning and end of the motion phase corresponding to toe-off and initial contact, respectively. In the case of the Parkinsonian gait cycle, also shuffling steps or lifting of heel caused by shank trembling without forward movement are counted as motion phases. We introduce a control state Z, which controls the online search for a new motion phase within a single detected unrest phase. This occurs in case of a festination or non-alternating step sequences. In Figure 2, the gait-phase-detection algorithm is represented as a state machine.

The first phase of the algorithm estimates the foot orientation continuously from the recorded linear acceleration and angular rates *a*(*t*), ω(*t*) ∈ **R**^3^ and extracts the Euler angles ϕ(*t*) ∈ **R**^3^. As no magnetometer readings are used, slow drift in the heading is taking place. The orientation estimation algorithm is described in Seel and Ruppin (34). Using the foot orientation quaternion, the linear acceleration and angular velocity vectors are transformed from the intrinsic measurement frame of the inertial sensor in the global coordinate system. The transformed vectors are denoted as $a_{g}\left(t\right),\omega_{g}\left(t\right)\in {\text{R}}^{3}$, respectively.

Next, the offsets in the aforementioned vectors are estimated during the rest phase using a moving average window of size *n*_d_ = 10 and subtracted from the vectors. The detrended vectors are denoted as $a_{\text{d,g}}\left(t\right),\omega_{\text{d,g}}\left(t\right)\in {\text{R}}^{3}$. From the extracted Euler angle, the initial angles are estimated during the rest phase using the previous moving average filter method. These angles are then subtracted from the Euler angles ϕ(*t*). The resulting angle vector is denoted as $\phi_{\text{d}}\left(t\right)\in {\text{R}}^{3}$. The pitch angle ϕ_d, pitch_ is defined as positive when the heel is above the toes and negative when the heel is below the toes.

#### 2.1.1. Detecting Rest and Unrest Phase

The transition between rest and unrest phases is detected using a threshold-based approach. If the Euclidean norm of the linear acceleration and angular velocities (∥*a*_d,g_(*t*)∥_2_ < *a*_rest_) ∧ (∥ω_d,g_(*t*)∥_2_ < ω_rest_) lie below the defined upper bounds *a*_rest_, ω_rest_ ∈ **R**_>0_ for at least *n*_*r*_ ∈ **N**_>0_ consecutive samples, then the algorithm will transition to a rest phase. Analogously, the unrest phase will be detected if the previous defined norms exceed the predefined thresholds (condition A).

#### 2.1.2. Detecting the Start of a Motion Phase

The motion phase is characterized by changes in foot orientation or displacement relative to the last rest phase. The algorithm exploits the pitch and roll angles of the foot to detect the start of a motion phase. During forward movement of the foot, starting from heel-off an increase in the foot pitch angle is observed. The pitch angle reaches a local maximum before the swing phase on toe-off (assuming a healthy gait cycle). Detecting a maximum in the pitch angle of the foot ϕ_d,itch_ > ϕ_p_, that is greater than a defined lower bound ϕ_p_ ∈ **R**, is the first condition B for detecting the start of the motion phase.

The alternative condition B is based on the roll angle ϕ_d,roll_ ∈ **R** and the acceleration vector. If a correlation in the maxima of roll angle and filtered acceleration norm is detected (both maxima are at most 0.25s time-displaced), then a motion phase is detected. The acceleration norm is filtered by means of a moving average filter of window size *n*_a_ of seven samples to discard less prominent local maxima caused by noise.

#### 2.1.3. Detecting the End of the Motion Phase

The end of the motion phase is characterized by an abrupt change of the acceleration as the cause of the initial contact of the foot with the ground. This is indicated by a large norm in the jerk signal *j*(*t*) ∈ **R**. However, a large jerk can also be observed at the start of the motion phase. Therefore, the initial contact can only be detected, after a defined time *t*_mot_ ∈ **R**_>0_ since the start of the motion phase has elapsed.

The jerk norm $j\left(t\right)={\left|\left|\frac{d\left(a_{\text{d,g}}\left(t\right)\right)}{dt}\right|\right|}_{2}$ is monitored from the beginning of the motion phase *t*_0_ ∈ **R**_>0_. The algorithm detects the end of the motion phase, if a large jerk norm exceeds the maximal registered jerk norm by a factor α ∈ **R**_>0_ during the gait cycle and if the horizontal velocity components are a factor β ∈ **R**_>0_ less than the registered maxima during the motion phase. These sub-conditions exploit that the velocity in the x-y transverse plane (forward and sideward movement) reaches a maximum velocity during the mid-motion phase and then decreases until the end of the motion phase. The parameter β ∈ **R**_>0_ is a factor that determines the threshold as a function of the maximum velocity during the motion phase. The velocity *v*(*t*) ∈ **R**^3^ is calculated using strap-down integration of the acceleration vector *a*_d,g_ during the motion phase.


$$\begin{array}{l} D_{1}:j\left(t\right)>\alpha \cdot \underset{\tau =\left[t_{0},t\right]}{\max}\left\{j\left(\tau \right)\right\}\\ D_{2}:|v_{\text{x}}\left(t\right)|<\beta \cdot \underset{\tau =\left[t_{0},t\right]}{\max}\left\{\left|v_{\text{x}}\left(\tau \right)\right|\right\}\wedge |v_{\text{y}}\left(t\right)|<\beta \cdot \underset{\tau =\left[t_{0},t\right]}{\max}\left\{\left|v_{\text{y}}\left(\tau \right)\right|\right\},\\ \text{ for }t>t_{0}+t_{\text{mot}} \end{array}$$

**Figure 3:**
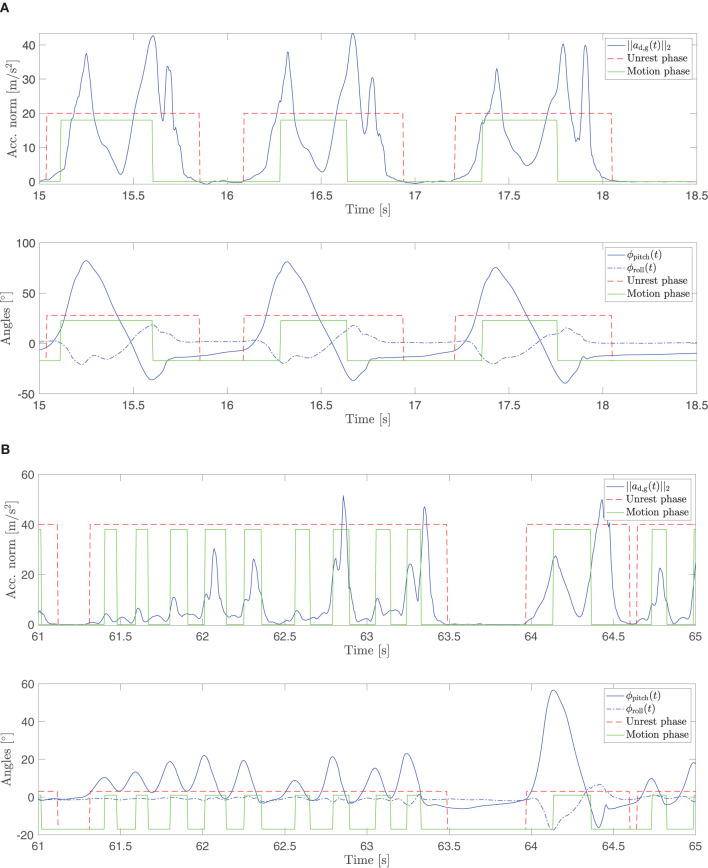
Examples of normal **(A)** and pathological **(B)** gait cycles and the corresponding acceleration norm, roll, and pitch angles. In **(B)**, a shank trembling episode during turning is displayed. During this episode, no rest phases are detected between motion phases. Furthermore, the changes in the pitch angle are smaller and more frequent than during normal walking.

Furthermore, the end of the motion phase is also characterized by a minimum in the pitch angle. At the start of the motion phase, the pitch angle reaches a local maximum. During the swing phase, the foot rotates in the opposite direction with the pitch angle decreasing until initial contact. Alternative to the previous condition, if a minimum in the pitch angle is found during the motion phase, assuming the roll angle is below a defined bound |ϕ_roll_| < ϕ_r_ and |ϕ_pitch_(*t*)| < κϕ_pitch,max_, then the end of the motion phase is detected. The parameter κ ∈ **R** determines the upper bound of the local minimum of the pitch angle for detecting the end of the motion phase (condition E). The two sub-conditions should prevent the algorithm from getting stuck in a local minimum before the initial contact. Figure 3 displays examples of normal and Parkinsonian gait cycles, respectively.

#### 2.1.4. Reactivation of State Z

As already mentioned above, the state Z controls the start of the search for the next motion phase. Initially, the state Z is active when entering the unrest phase. It deactivates at the end of each motion phase. In case that no rest phase is detected after a completed motion phase within the interval *t*_*x*_ ∈ **R**_>0_, then the following conditions are validated:


$$\begin{array}{l} F:\phi_{\text{pitch}}\left(t\right)|<p_{1}\wedge |\phi_{\text{roll}}\left(t\right)|<p_{1} \end{array}$$

$$\begin{array}{l} G:\text{var}\left(||a\left(t\right)|{|}_{2}\right)<p_{2} \end{array}$$

If any of the above conditions is valid for at least *n*_*r*_ ∈ **N**_>0_ consecutive samples or if the local maximum in the pitch angle is larger than a threshold ϕ_thres_ ∈ ℝ (condition H), then the state Z is reactivated. The same fixed parameters are used for the gait-phase-detection algorithm for all patients and are listed in Table 1.

**Table 1:** Parameter values used in the gait-phase-detection algorithm.

**Parameter**	**Value**	**Parameter**	**Value**
*a*_rest_	0.5 m/s^2^	α	1.2
*w*_rest_	0.11 rad s^1^	β	0.6
Δ	0.25 s	κ	0.8
*n*_r_	10	*p*_1_	0.75 °/s
ϕ_p_	1 °	*p*_2_	1.5 m/s^2^
ϕ_r_	5 °	ϕ_thres_	15 °
*a*_s_	2 m/s^2^	*t*_mot_	0.075 s
*t*_x_	0.1 s	*n*_d_	10
*n*_a_	7		

### 2.2. Feature Extraction

For detecting FoG-affected motion phases, we employ machine learning methods. As inputs for the classification, spatio-temporal gait and signal features are extracted from the recently completed motion phase. Ten features are considered that capture the dynamics, range, and kind of foot motion:

- Maximum of the foot acceleration norm,
- Maximum of the pitch angle,
- Minimum of the pitch angle,
- Stride length,
- Maximum gait velocity,
- Turning angle,
- Turned flag {0, 1},
- Maximum turn rate,
- Average turn rate, and
- Step duration.

The stride length and gait velocity are calculated using strap-down integration. At the end of each motion phase, a correction of the estimated velocities is carried out, based on the constraint that the velocities at the end of the motion phase are zero. The offset from the zero line is compensated from the velocity vector and the integrated part of the offset in the x-y plane is removed from the stride length. The angles are obtained from the orientation estimation that has been introduced in section 2.1. In addition, a turning detection method was developed based on the jaw (heading) angle of the foot. A monotonous increase or decrease of this angle without zero crossings during a motion phase indicates a turning action (turned flag set to one). The turning angle corresponds to the yaw angle at the end of the motion phase.

In order to investigate the importance of the features, we employed an univariate feature ranking based on the chi-square test. The negative logarithm of the *p*-values of the chi-square test is used as the importance value. Importance values above 1.3 indicate a *p*-value below 0.05 so that the feature is relevant for machine learning.

### 2.3. Classification

In order to differentiate between FoG-affected and normal motion phases, three different classifiers are trained, a SVM classifier using all features with *p* < 0.05 of the chi-square test, another SVM classifier using the five most important features with *p* < 0.05, and an AdaBoost classifier using all features with *p* < 0.05. For the hyperparameter tuning, a Bayesian optimisation was carried out.

First, classifiers were trained on the features of the actual motion phase (classifiers *C*_0_). To investigate whether information from previous gait cycles can improve the detection of FoG-affected motion phases, new classifiers were trained with additional information from the two preceding motion phases (classifiers *C*_−2,−1,0_). Furthermore, we investigated whether a prediction of the next motion phase type (FoG or not) could be made solely based on the features from the two preceding motion phases (classifiers *C*_−2,−1_). For the classification training and validation, we used the free software machine learning library scikit-learn (version 0.24.2) for Python (version 3.8.2).

### 2.4. Data Set

The data set used for training the model was recorded at the Department of Neurology, Charité—Universitätsmedizin Berlin. Idiopathic Parkinson's patients, who showed Freezing of Gait episodes, were recruited for the study. The performed trials were reviewed and approved by the ethics committee of the Charité–Universitätsmedizin Berlin. Written consent was obtained from all subjects. The age range of the 16 recruited patients was between 50 and 82 years (68 ± 9.2 years). The recruited population consisted of 13 male and three female patients, out of the population seven patients had a DBS implant. Patients were recorded in individual conditions which revealed FoG (medication on/off; DBS patients: stimulation on/off). The scores of the Unified Parkinson's Disease Rating Scale Part III (UPDRS-III) were collected and ranged between 18 and 64, including patients with mild and moderate motor impairments (32.9±14.1).

Each patient performed two trials of the freezing assessment course proposed by Ziegler et al. (11). This clinical test consists of several scenarios, which can potentially trigger FoG episodes. The patients start from a sitting position, stand up and walk 1 m, followed by two 360° turns in both directions within a marked square (40 × 40 cm). Afterwards, the patient walks to the door, opens the door, and leaves the room. The last part of the test, after leaving the room, consists of reentering the room and returning to the sitting position. Two synchronized inertial sensors (MUSCLELAB^TM^, Ergotest Innovation AS, Oslo, Norway) were used to record 3D acceleration and rates of turn at 200 Hz. The sensors were positioned at the back of the foot using straps (cf. Figure 1). Each trial was video-recorded from two perspectives. A custom-built, battery-powered portable device was used to enable synchronization of the inertial-sensor data and the videos. Synchronous vibration, light, and sound signals are generated at a push of a button. The device is held up to one of the inertial sensors at the beginning and end of each trial and the button is pressed. The vibrations, visible in the inertial sensor, and the sounds and light flashes, present in the video, are used for the synchronization.

The trials were subsequently evaluated by two clinical experts based on the recorded video data. The start, end, and duration of each FoG episode were annotated. Annotated FoG episodes were associated with the occurrence of festination, shuffling, shank trembling, and akinesia. The gait phase detection and the extraction of the features were done offline in post-processing using Matlab/Simulink 2021a (The Mathworks Inc., USA).

In total, 2,621 foot motion phases during gait were recorded from which 1,750 were marked as FoG. The correlation between the annotations of the experts was higher than 80% for all patients and on average 93.7%±7.7%. The correlation is based on the detected motion phases. Therefore, the *AND*-combination and an *OR*-combination of the two labels were calculated. The ratio of *AND* FoG motion phases to *OR* FoG motion phases then served as a measure of the correlation between the two experts' annotations. The annotated FoG episodes are not foot side but gait specific. For this reason, the classifiers were trained using the features and derived labels from both feet.

### 2.5. Validation Method

The classification problem consists of classifying the motion phases in FoG-affected and normal motion phases. As ground truth, we took the OR-combination of the labels provided by the two experts. A motion phase was labeled as a normal motion phase in case none of the experts annotated a FoG episode at the end time of the motion phase, otherwise the label was set to FoG. The models were evaluated using leave-one-patient-out cross-validation, to validate the performance of the models in generalizing on unseen patient's data. During cross-validation, the classifiers were validated in a single patient for both feet independently, while they were trained using motion phases from all other patients and both feet. The classifier performance measures considered for the evaluation are sensitivity, specificity, accuracy, and Area Under the Curve (AUC). The significance of the performance differences between the classifier models was investigated by comparing the mean and the standard deviation of AUC based on the statistical t-test. We used a significance level of *p* < 0.05.

## 3. Results

Table 2 displays the features sorted by their importance. All selected features displayed a *p*-value smaller than 0.05 for the chi-square test. The optimized hyperparameters are listed in the Tables 3, 4. In Table 5, the results of the leave-one-patient-out cross-validation for all trained classifiers are summarized.

**Table 2:** Feature ranking based on the chi-square test.

**Features**	**–log(p-value)**
Maximum gait velocity	527.8
Step duration	501.8
Stride length	485.8
Maximum of the pitch angle	394.2
Minimum of the pitch angle	206.6
Average turn rate	71.4
Turned flag {0, 1}	43.7
Maximum of the foot acceleration norm	31.3
Turning angle	29.3
Maximum turn rate	17.5

**Table 3:** SVM hyperparameters.

**Classifiers**		**Hyperparameters**		
		**Kernel**	**C**	**Gamma**
*C*_0_ (Trained on the actual motion phase)	SVM_10	RBF	5.9	0.005
	SVM_5	RBF	0.032	0.33
*C*_−2,−1,0_ (Trained on the actual and two preceding motion phases)	SVM_10	RBF	2.37	0.026
	SVM_5	RBF	10	0.006
*C*_−2,−1_ (Predicting model)	SVM_10	RBF	10	0.006
	SVM_5	RBF	10	0.081

**Table 4:** AdaBoost hyperparameters.

**Classifiers**		**Hyperparameters**				
		**Weak learner**	**Criterion**	**max.** **Depth**	**No.** **estimators**	**Learning** **rate**
*C*_0_ (Trained on the actual motion phase)	AdaBoost	Decision tree	Entropy	3	20	0.318
*C*_−2,−1,0_ (Trained on the actual and two preceding motion phases)	AdaBoost	Decision tree	Gini	2	20	0.447
*C*_−2,−1_ (Predicting Model)	AdaBoost	Decision tree	Entropy	5	20	0.01

**Table 5:** Overview of the leave-one-patient-out cross- validation results for all classifiers taking both feet into account.

**Classifier**		**Spec**.	**Sens**.	**Acc**.	**AUC**
*C*_0_ (Trained on actual motion phase)	SVM_10	80.2% ±10.4%	85.8% ±8.0%	84.6% ±6.0%	90.2% ±6.8%
	SVM_5	76.5% ±11.0%	85.4% ±7.3%	83.5% ±5.1%	86.1% +7.7%
	AdaBoost	78.8% ±11.9%	85.5% ±8.6	84.0% ±7.0%	87.5% ±8.2%
*C*_−2,−1,0_ (Trained on actual and two preceding motion phases)	SVM_10	83.3% ±17.1%	88.5% ±5.8%	88.6% ±5.4%	92.8% ±5.9%
	SVM_5	82.6% ±17.5%	86.4% ±8.3%	87.1% ±5.2%	92.3% ±5.6%
	AdaBoost	80.8% ±15.7%	88.3% ±7.1%	87.2% ±5.7%	90.5% ±6.6%
*C*_−2,−1_ (Predicting model)	SVM_10	70.3% ±18.4%	81.6% ±7.7%	80.5% ±5.0%	82.8% ±7.1%
	SVM_5	70.3% ±18.7%	80.8% ±9.1%	79.6% ±4.9%	80.9% ±9.1%
	AdaBoost	69.2% ±17.2%	80.1% ±10.9%	79.3% ±6.1%	80.3% ±9.7%

In case of the classifiers *C*_0_ trained on the actual motion phase, the trained SVM model with all 10 features (SVM_10) performed on average over all patients with 80.2, 85.8, 84.6, and 90.2% for specificity, sensitivity, accuracy, and AUC, respectively. The trained SVM model with the five most important features (SVM_5) performed on average with 76.5, 85.4, 83.5, and 86.1% for specificity, sensitivity, accuracy and AUC, respectively. Finally, the AdaBoost model achieved performance values of 78.8, 85.5, 84.0, and 87.5% on average for specificity, sensitivity, accuracy, and AUC, respectively. Regarding the performance values, the model SVM_10 outperformed the other two classifiers. Despite that, no statistical difference between the three *C*_0_ classifiers could be observed based on the *t*-test. In Table 6, the results for each patient and each body side are displayed for the model SVM_10 with features from the actual motion phase.

**Table 6:** Results of the SVM classifier *C*_0_ for each patient trained on all 10 features of the actual motion phase (SVM_10).

	**Left foot**				**Right foot**				**Expert** **corr**.	**More aff.** **side**
**Patient**	**Spec**.	**Sens**.	**AUC**	**FoG/** **Normal**	**Spec**.	**Sens**.	**AUC**	**FoG/** **Normal**		
S1	81.3%	86.2%	90.7%	32/29	**82.8%**	**96.2%**	**96.2%**	29/26	93.8%	left
S2	54.2%	86.1%	76.7%	24/108	**63.6%**	**76.9%**	**77.6%**	22/78	84.5%	x
S3	**70.6%**	**82.0%**	**79.7%**	17/61	73.3%	59.3%	66.4%	15/54	93.4%	x
S4	81.8%	95.7%	92.1%	77/23	**81.4%**	**85.7%**	**95.6%**	70/14	88.5%	right
S5	80.0%	78.6%	83.4%	25/14	**89.7%**	**62.5%**	**89.7%**	29/16	100%	left
S6	84.0%	81.4%	91.9%	25/59	**95.0%**	**86.0%**	**97.4%**	20/57	93.0%	left
S7	**77.3%**	**100%**	**98.3%**	22/24	82.6%	89.7%	93.9%	23/29	93.0%	left
S8	**100%**	**80.6%**	**98.9%**	26/31	96.0%	88.6%	96.1%	25/35	79.5%	left
S9	**90.5%**	**100%**	**98.1%**	21/60	69.2%	95.4%	94.7%	26/65	97.0%	left
S10	84.8%	88.6%	94.2%	33/35	**90.0%**	**84.4%**	**94.4%**	30/32	95.5%	x
S11	63.6%	93.0%	88.2%	33/128	**85.2%**	**91.2%**	**90.8%**	27/113	97.5%	left
S12	**95.2%**	**86.3%**	**96.7%**	21/51	87.0%	89.7%	95.4%	23/29	98.5%	left
S13	**82.4%**	**75.0%**	**89.1%**	17/64	66.7%	74.1%	82.6%	21/54	96.2%	left
S14	**83.3%**	**92.3%**	**95.8%**	24/39	90.9%	83.8%	91.6%	22/37	100%	x
S15	**61.5%**	**95.5%**	**87.3%**	26/176	61.9%	87.4%	86.0%	21/175	85.2%	right
S16	**90.9%**	**87.5%**	**92.9%**	22/16	82.6%	83.3%	89.9%	23/18	100%	left
Mean	80.1%	88.0%	90.9%		81.1%	83.4%	89.9%			
Std. dev.	12.4%	7.5%	6.6%		10.9%	10.5%	8.3%			

*The bold values correspond to the body side with the better results in AUC. In the column ‘FoG/Normal' the ratio of marked normal motion phases to annotated FoG motion phases is listed. The results from the correlation analysis between the two experts and the most affected side (derived from the UPDRS, x stands for unknown) are shown in the last two columns*.

The SVM classifier *C*_−2,−1,0_, trained with 10 features of each of the actual and two preceding motion phases (SVM_10), outperformed the models *C*_0_ only trained with information from the actual motion phase. Here, 88.5% sensitivity, 83.3% specificity, 88.6% accuracy, and 92.8% AUC could be achieved. The other *C*_−2,−1,0_ classifiers, SVM_5 and AdaBoost, show slightly decreased performance values compared to the SVM_10 classifier *C*_−2,−1,0_ for which the results of each patient and foot are shown in Table 7. Again, no statistical difference could be observed based on the *t*-test between all *C*_−2,−1,0_ and *C*_0_ classifiers with respect to the AUC values.

**Table 7:** Result of the SVM classifier *C*_−2,−1,0_ for each patient trained with all 10 features of the actual and the two preceding motion phases (SVM_10).

	**Left foot**				**Right foot**				**Expert** **corr**.	**More aff.** **side**
**Patient**	**Spec**.	**Sens**.	**AUC**	**FoG/** **Normal**	**Spec**.	**Sens**.	**AUC**	**FoG/** **Normal**		
S1	89.3%	82.8%	91.7%	32/29	**84.0%**	**96.2%**	**97.4%**	29/26	93.8%	left
S2	**65.2%**	**89.7%**	**85.7%**	24/108	66.7%	76.6%	84.4%	22/78	84.5%	x
S3	60.0%	82.0%	78.7%	17/61	**38.5%**	**85.2%**	**80.1%**	15/54	93.4%	x
S4	87.3%	87.0%	93.6%	77/23	**92.3%**	**84.6%**	**96.0%**	70/14	88.5%	right
S5	90.9%	76.9%	94.8%	25/14	**92.0%**	**87.5%**	**96.5%**	29/16	100%	left
S6	**95.8%**	**82.8%**	**95.5%**	25/59	84.2%	85.7%	90.4%	20/57	93.0%	left
S7	100.0%	87.5%	98.3%	22/24	**95.2%**	**89.7%**	**98.4%**	23/29	93.0%	left
S8	**100.0%**	**86.2%**	**99.1%**	26/31	100.0%	79.4%	95.1%	25/35	79.5%	left
S9	**95.2%**	**100.0%**	**100.0%**	21/60	88.0%	93.5%	96.9%	26/65	97.0%	left
S10	90.3%	87.9%	94.2%	33/35	96.4%	80.0%	94.0%	30/32	95.5%	x
S11	63.3%	96.1%	86.6%	33/128	**76.0%**	**96.4%**	**89.0%**	27/113	97.5%	left
S12	94.7%	95.9%	98.6%	21/51	**95.2%**	**100.0%**	**99.6%**	23/29	98.5%	left
S13	**93.3%**	**81.3%**	**95.0%**	17/64	65.0%	83.0%	83.9%	21/54	96.2%	left
S14	**95.0%**	**92.3%**	**96.0%**	24/39	94.4%	83.8%	94.1%	22/37	100%	x
S15	**42.3%**	**97.1%**	**88.2%**	26/176	47.6%	91.2%	85.5%	21/175	85.2%	right
S16	95.0%	92.9%	98.9%	22/16	**95.2%**	**100.0%**	**99.1%**	23/18	100%	left
Mean	84.9%	88.6%	93.4%		81.9%	88.3%	92.5%			
Std. dev.	17.2%	6.6%	5.9%		18.4%	7.3%	6.2%			

*The bold values correspond to the body side with the better results in AUC. In the column ‘FoG/Normal' the ratio of marked normal motion phases to annotated FoG motion phases is listed. The results from the correlation analysis between the two experts and the most affected side (derived from the UPDRS, x stands for unknown) are shown in the last two columns*.

We also trained classifier models *C*_−2,−1_ for predicting whether the next motion phase is normal or FoG-affected based on features from the two preceding motion phases. The best performance measures were 70.3% specificity, 81.6% sensitivity, and 82.8% AUC for the SVM_10. The AUC performance of all *C*_−2,−1_ classifiers (FoG prediction models) was significantly lower in comparison to the *C*_0_ and *C*_−2,−1,0_ classifiers (FoG detection models) based on the *t*-tests (*p*>0.05) except for *C*_0_-SVM_5.

We took a closer look at the motion phases where transitions (beginning or end of FoG episodes) occurred and evaluated them separately from the other motion phases. The SVM model *C*_−2,−1_ with all features from the last two preceding motion phases (SVM_10) could not detect the transitions well in advance. The results were only 27.4% specificity, 38.9% sensitivity, and 33.1% accuracy.

In Table 8, the results of the classifiers *C*_0_, *C*_−2,−1,0_, and *C*_−2,−1_ (trained on both feet) are listed for the validation at the left and right foot and a chosen best foot side. The criterion for choosing the best side was the AUC, i.e., the foot side with the highest AUC for each patient was chosen as the best side for FoG detection or prediction. The average AUC performance values increased for the best foot classifiers compared to the left and right foot classifiers, but not statistically significant.

**Table 8:** Overview of the average results for all classifiers (trained on both feet) using the left foot, right foot, or best foot for validation.

**Classifiers**		**Left foot**			**Right foot**			**Best foot**		
		**Spec**.	**Sens**.	**AUC**	**Spec**.	**Sens**.	**AUC**	**Spec**.	**Sens**.	**AUC**
*C*_0_	SVM_10	80.1% ±12.3%	88.0% ±7.5%	90.9% ±6.6%	81.1% ±10.9%	83.4% ±10.5%	89.9% ±8.3%	83.7% ±10.9%	86.4% ±9.8%	92.4% ±6.4%
	SVM_5	76.0% ±12.5%	86.6% ±9.6%	85.7% ±8.9%	77.5% ±11.9%	84.2% ±7.1%	86.8% ±7.6%	80.3% ±10.8%	85.6% ±8.6%	88.5% ±8.1%
	AdaBoost	80.1% ±11.4%	86.9% ±10.4%	88.8% ±7.4%	77.8% ±14.5%	84.0% ±12.0%	86.6% ±11.9%	82.4% ±11.8%	88.8% ±7.5%	91.6% ±5.9%
*C*_−2,−1,0_	SVM_10	84.9% ±17.2%	88.6% ±6.6%	93.4% ±5.9%	81.9% ±18.4%	88.3% ±7.3%	92.5% ±6.2%	84.1% ±19.2%	91.0% ±6.4%	94.4% ±5.7%
	SVM_5	85.0% ±17.7%	86.4% ±8.8%	93.6% ±5.4%	80.6% ±19.7%	86.2% ±9.4%	91.3% ±6.2%	84.5% ±17.2%	87.2% ±10.0%	93.9% ±5.5%
	AdaBoost	80.2% ±17.6%	89.6% ±6.8%	91.9% ±5.3%	81.9% ±14.8%	86.9% ±8.8%	89.7% ±8.6%	83.6% ±14.6%	89.7% ±6.5%	92.8% ±5.6%
*C*_−2,−1_	SVM_10	69.3% ±20.8%	81.5% ±9.6%	82.9% ±7.9%	71.6% ±17.2%	81.5% ±7.3%	82.8% ±6.6%	72.6% ±18.6%	83.2% ±7.6%	84.7% ±6.8%
	SVM_5	68.8% ±21.0%	81.2% ±10.5%	81.4% ±9.7%	72.0% ±17.2%	80.4% ±8.8%	80.8% ±9.3%	73.1% ±20.5%	81.4% ±8.9%	83.3% ±9.7%
	AdaBoost	67.9% ±18.4%	80.9% ±12.8%	79.6% ±11.7%	70.8% ±17.4%	79.5% ±11.7%	81.5% ±7.8%	71.1% ±17.3%	80.0% ±13.3%	82.9% ±8.2%

*The results of the classifiers C_0_ (trained on the actual motion phase), C_−2,−1,0_ (trained on the actual and two preceding motion phases), and C_−2,−1_ (predicting model) for all models are listed*.

## 4. Discussion

The aim of the adaptive gait-phase-synchronous cueing concept is to stimulate upon detection of a FoG motion phase for a defined time interval in order to improve the gait pattern and to prevent akinesia. Our hypothesis is that akinesia usually follows other FoG symptoms like shuffling, festination, and shank trembling. Based on the recorded data, we found that 97% of motion phases within a 3 s interval before akinesia were marked as pathological gait of these other subtypes. This indicates that akinesia rarely occurs suddenly and is mainly preceded by other FoG symptoms. This requires a reliable FoG detection in real-time with short latencies. To achieve this, we combined features from a gait-phase-detection algorithm suitable for Parkinsonian gait with several machine learning models and evaluated their performance in 16 PD patients, severely affected by FoG.

The results of our classification performance are not directly comparable to previous literature, because of several methodological differences. Most FoG detection algorithms use more than one IMU sensor and sensors at the ankle/shank or waist and not the feet. Another limitation for comparisons is that not all parameters are usually listed in publications, or the parameters are manually tuned for each patient (thresholds, hyperparameters, etc.). Therefore, it is difficult to determine whether the differences in performance are due to the methods or the data sets used.

Yet, our algorithm, despite its simple hardware configuration and low computational demands, matches the quality of previous research: The reported average classification performance in this article is comparable with the best results for patient-independent classifiers reported in the literature using multiple sensors or deep learning approaches. Naghavi et al. (24) achieved a slightly higher sensitivity (90.2% compared to our 88.5%) but evaluated the model only with seven PD patients. Borzì et al. (28) reported a slightly higher specificity (86.3% compared to our 83.3%) while our sensitivity was 3.3% higher. Sliding window-based approaches using IMU data from the waist with deep learning yielded a comparable performance in terms of accuracy (85–89%) compared to our approach (89%) but are computationally more demanding (26, 27). Other classifiers from the literature showed lower performance values (21–23, 25) or used patient-individual classifiers (29, 33).

The idea of this article, to rely solely on a single foot sensor for gait-phase and FoG detection, presents a new technical solution that could be very practicable. The proposed work provides an essential contribution toward the everyday use of gait monitoring technologies. Digital patient care is one central aspect of the ongoing transformation of medicine and society. A single sensor in the shoes could seamlessly integrate into the everyday life of patients.

The quality of the presented FoG detection algorithms, based solely on a foot sensor, is not shown in the literature. Moore et al. (21) investigated the usage of a 1D accelerometer at the foot to determine a freezing index from a sliding window using the 50 Hz sampled acceleration in walking direction. We applied this method to our data set for comparison and observed an AUC of about 80% on average for both feet for window sizes ranging from 2.5 s to 10 s (see Supplementary Material). These values are below the maximal AUC of 93% resulting from our approach. In addition, we determined the freezing index for all 3 axes of the accelerometer and gyroscope separately. Then, a common threshold was applied to all obtained freezing indexes, and FoG was robustly detected when the majority of axes indicated FoG. This approach was in analogy to Moore et al. (21) where the outcomes of several 1D accelerometer-based detectors at different body segments were fused to obtain a robust FoG detection. However, the obtained performance with the 6D approach on our data set did not differ from the 1D approach. Therefore, the performance gain with our machine-learning approach is likely to be explained by the use of gait features from fused 6D data. Another advantage of our sensor location is that gait phases are captured with high fidelity in severely affected patients. To allow benchmarking of algorithms by other research groups using sensors at feet, we include our data in open source (see Supplementary Material). We aim to compare our single sensor classification approach with others (including multiple sensors) using future open-access available data sets of PD gait with labeled FoG episodes. Currently, there is no such multi-segmental data set available with raw 6D inertial-sensor data from the feet.

The observed standard deviations for the performance values are relatively high and explain why no statistical differences in our classifier models have been observed except between some FoG detection and prediction models. A reason for that is the strict evaluation scheme chosen in this work, where the classification was evaluated on completely unknown patients. Another reason could be the distribution of the FoG subtypes through different patients. As patients had shown different severity of disease and variation of FoG symptoms, a leave-one-patient-out cross-validation would exclude a patient which showed a particular subtype from the training data set leaving at the worst-case scenario no representative samples for that subtype. In this case, collecting more data from new patients would help to solve the problem. Another problem is a strong misbalance of labels in an evaluated subject making the calculation of sensitivity or specificity error-prone. S15 is an example for such a misbalance. Of the few normal steps, only about half are correctly classified, giving a low value for specificity.

In this work, the classification was done after a completed motion phase. This results in a variable latency for the detection of FoG depending on the duration of the motion phase. From the available data set, it was calculated that the average motion phase duration was 0.7 s and maximal duration was 1.2 s. Therefore, in the worst-case scenario, the latency in detecting FoG was 1.2 s. FoG-affected motion phases are usually shorter in duration (average of 0.14 s) than normal motion phases. For this reason, the typical latency in motion-phase-based FoG detection will be lower than in sliding-window-based approaches with a fixed window size.

Comparing the two classifiers *C*_0_ and *C*_−2,−1,0_, the SVM_10 and AdaBoost models trained with the actual and two preceding motion phases showed an improvement of approximately 3% in all three performance measures. However, this is not statistically significant. Predicting whether the next motion phase is a FoG or a normal motion phase could be achieved with 70.3% specificity, 81.6% sensitivity, 80.5% accuracy, and 82.8% area under the curve. Upon further analysis, we found out that transitions from normal to pathological motion phases and vice versa can only be detected with a very low accuracy. For this reason, a reliable prediction of the onset of FoG episodes could not be achieved.

The SVM models with all features of the segmented motion phases performed on average better than the SVM models with the reduced feature set and the AdaBoost models. However, no statistical difference could be found between all models.

The SVM models with all features for the segmented motion phases performed on average better than the SVM models with the reduced feature set and the AdaBoost models although this was not statistically significant. For implementation on embedded systems, the AdaBoost classifiers are presumably better suitable as the number of parameters and consequently the memory demand are significantly lower: the SVM_10 classifier *C*_0_ with 10 features has 10,925 and the AdaBoost classifier *C*_0_ only 180 floating-point parameters.

Finally, the two feet of each subject yielded different FoG detection results. It seems favorable to search for the more suitable leg with higher classification performance when using only a single sensor in an automated cueing system. The performance boost will be up to about 3% for *C*_0_ on average but can be up to 13% in an individual patient when using a specific leg (see, e.g., S3 in Table 6). We could not identify a clear correlation between the results of the classifiers and the most affected side, which was annotated by the clinical experts using the UPDRS. Therefore, we recommend testing both feet in an individual patient to choose the best foot based on the observed classification performance.

Limitations of the study are the limited number of 16 PD patients and the sole use of clinical data although the used test by Ziegler et al. (11) aims at triggering FoG by using situations relevant in everyday life such as gait initiation, turning, walking through doors, and terminating gait. Currently, our algorithm assumes that the person is standing up, standing, walking, or sitting down. Further developments should detect these conditions automatically to avoid false detections during other activities like sitting.

Starting hesitation and akinesia often show no foot motion phases at all and therefore cannot be detected as FoG symptoms with our motion phase-centered approach. This is a limitation of the presented method. To assist the patient in unfreezing from such motor blockades, we suggest to offer rhythmic cueing which can be triggered by the patient.

At the moment, we implement some of the presented classifier models in an on-demand cueing system for gait-phase-synchronous electrical stimulation (35). The algorithms will be distributed onto the hardware: Gait phase detection and feature extraction are running on the foot sensor and the classifier on the electrical stimulator. We plan to evaluate the real-time detection system and the adaptive cueing concept in a clinical trial.

## 5. Conclusions

We propose new methods for real-time detection of gait phases and FoG in Parkinsonian gait using a wearable sensor at the shoe. The novel Parkinsonian gait-phase-detection algorithm differentiates three phases, namely rest, unrest, and motion of the foot, and does not fail during FoG gait patterns such as shuffling, festination, and shank trembling. Thereupon, machine-learning-based methods for detecting FoG motion phases were developed. For the first time, spatio-temporal gait and signal features have been used as inputs to the classifier models. An SVM model, trained only with 10 features from the actual motion phase, already yielded 85.8% sensitivity, 80.2% specificity, 84.6% accuracy, and 90.2% AUC. By using in total 30 features from the actual and two preceding motion phases, we could improve the above results by approximately 3% on average, but not statistically significant. The observed prediction performance remains behind the observed detection performance. Our work paves the way for on-demand, gait-synchronous cueing in PD patients suffering from freezing.

## Data Availability Statement

The original contributions presented in the study are included in the article/Supplementary Material, further inquiries can be directed to the corresponding author.

## Ethics Statement

The studies involving human participants were reviewed and approved by the Ethics Committee of the Charité Universitätsmedizin Berlin. The patients/participants provided their written informed consent to participate in this study.

## Author Contributions

AD developed the methods proposed in the paper and wrote the initial draft of the manuscript. Latter was revised by all remaining authors. VW helped with the feature extraction and solving the classification problem. TS advised on the developing process. MJ, CS-H, JM-O, and NW conducted the tests with Parkinson's patients in clinic, as well as data recording and annotating. AK, TS, and NW performed funding acquisition and supervision. All authors contributed to the article and approved the submitted version.

## Funding

This work was funded by the German Federal Ministry of Education and Research (BMBF) within the project Mobil4Park (FKZ 16SV8168). We acknowledge support by the German Research Foundation and the Open Access Publication Fund of TU Berlin. NW is a Freigeist-Fellow supported by the Volkswagen Foundation, and a participant in the BIH Charité Clinician Scientist Program. AK and NW were supported by funding of the German Research Foundation in the framework of the TRR 295: Retuning dynamic motor network disorders using neuromodulation (Project No. 424778381).

## Conflict of Interest

AD is employee and TS is co-funder of the SensorStim Neurotechnology GmbH, which is a company developing sensor-based stimulation devices. The remaining authors declare that the research was conducted in the absence of any commercial or financial relationships that could be construed as a potential conflict of interest.

## Publisher's Note

All claims expressed in this article are solely those of the authors and do not necessarily represent those of their affiliated organizations, or those of the publisher, the editors and the reviewers. Any product that may be evaluated in this article, or claim that may be made by its manufacturer, is not guaranteed or endorsed by the publisher.
