# Supplementary material for: Real-Time Detection of Freezing Motions in Parkinson's Patients for Adaptive Gait Phase Synchronous Cueing
Source: Front Neurol. 2021 Dec 6;12:720516. doi: 10.3389/fneur.2021.720516 (PMC8685223; doi:10.3389/fneur.2021.720516)
Supplement: Supplementary file 1 [file Data_Sheet_1.pdf]

Supplementary Material

1 SUPPLEMENTARY DATA

The raw inertial data (3D accelerometer and 3D gyroscope, sampled at 200 Hz) are provided as CSV files for all subjects and both feet together with the FoG labels. The corresponding files for the subjects S1 to S16 and the left and right foot are compressed in a single zip archive and named as follows:
`S1_left_foot_trial_1.csv`, `S1_right_foot_trial_1.csv`,
`S1_left_foot_trial_2.csv`, `S1_right_foot_trial_2.csv`,
...
`S16_left_foot_trial_1.csv`, `S16_right_foot_trial_1.csv`,
`S16_left_foot_trial_2.csv`, `S16_right_foot_trial_2.csv`.
Every subject conducted two trials. The first column is the time vector in seconds. Columns two to four contain the 3D-accelerations (x-, y-, and z-axis) in m/s^2, and columns five to seven contain the 3D angular velocities of the gyroscope (x-, y-, and z-axis) in deg/s (see Figure 1 in the article for sensor placement). The last column represents the FoG label from the experts ($1 \triangleq$ FoG active).

2 SUPPLEMENTARY TABLES AND FIGURES

The sliding-window-based algorithm by Moore et al. (2013) for FoG detection has been applied to our data set for comparison. For each of the six axes (three accelerations and three angular velocities) of the inertial sensor, a Freezing Index (FI) signal was determined as the ratio of the power (area under the curve) in the freeze (3-8 Hz) and locomotor (0-3 Hz) bands from the power spectrum of the sliding signal window. FoG is detected for a single axis when the FI exceeds a defined threshold (single axis version) or when the majority of axes indicate FoG for a common threshold (6D version). Figures S1 and S2 show the accuracy depending on the window size and threshold as well as the corresponding Receiver Operating Characteristic (ROC) curve for the left and right foot, respectively. The curves represent means over all subjects. For four selected window sizes, Table S1 reports the Area Under Curve (AUC) values and maximal achievable accuracies for the single axis detection version and the fused 6D detection version. Values have been averaged over all subjects and both feet.

2.1 Tables

Table S1. Average rating of the FoG detection performance (maximum accuracy and AUC) of all subjects and both feet for different window sizes using the approach by Moore et al. (2013). Numbers are reported for the usage of different single sensor axes of the 3D accelerometer and 3D gyroscope as well as for the fusion of freezing indexes from all axes (6D version).

	Window size							
	2.5 s		5 s		7.5 s		10 s	
Data \ Rating	Max. acc.	AUC	Max. acc.	AUC	Max. acc.	AUC	Max. acc.	AUC
Acc. x	0.75	0.80	0.76	0.82	0.75	0.81	0.75	0.80
Acc. y	0.75	0.80	0.76	0.82	0.76	0.81	0.75	0.81
Acc. z	0.73	0.80	0.74	0.81	0.73	0.80	0.73	0.79
Gyr. x	0.74	0.80	0.76	0.81	0.75	0.80	0.74	0.79
Gyr. y	0.72	0.78	0.73	0.79	0.73	0.78	0.73	0.78
Gyr. z	0.73	0.78	0.75	0.80	0.74	0.79	0.74	0.78
6D	0.75	0.80	0.75	0.80	0.74	0.80	0.74	0.79

2.2 Figures

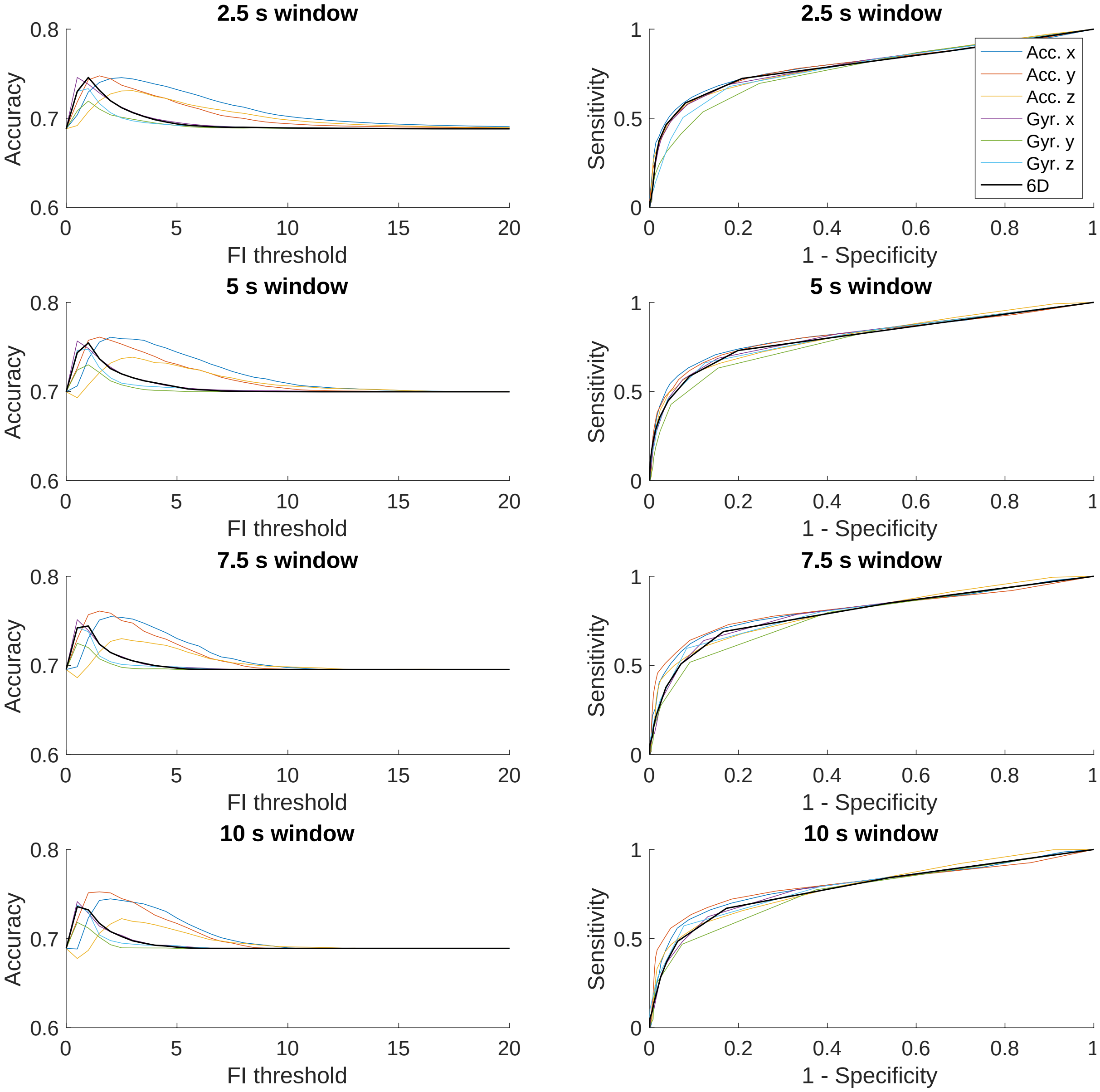

Figure S1. FoG detection with the window-based approach by Moore et al. (2013) using the left foot sensor. Average accuracy, as function of the FI threshold, and ROCs for four different window sizes and used inertial sensor axes (single axis, 6D) are shown. The relation of sensitivity and specificity can be examined in the ROC curves (right column of plots). Averaging was performed over all 16 subjects.

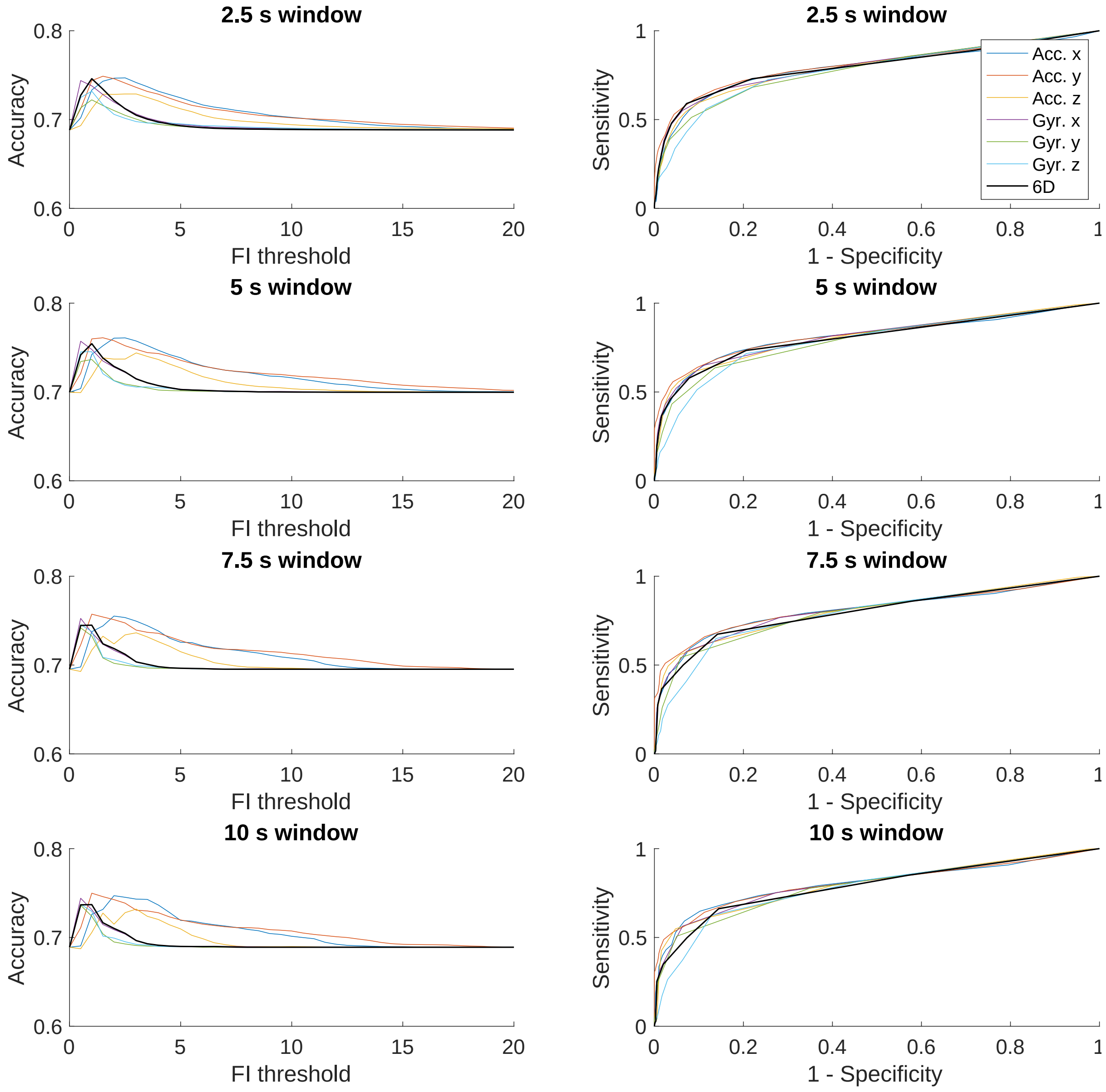

Figure S2. FoG detection with the window-based approach by Moore et al. (2013) using the right foot sensor. Average accuracy, as function of the FI threshold, and ROCs for four different window sizes and used inertial sensor axes (single axis, 6D) are shown. The relation of sensitivity and specificity can be examined in the ROC curves (right column of plots). Averaging was performed over all 16 subjects.

REFERENCES

Moore, S. T., Yungher, D. A., Morris, T. R., Dilda, V., MacDougall, H. G., Shine, J. M., et al. (2013). Autonomous identification of freezing of gait in Parkinson's disease from lower-body segmental accelerometry. *Journal of NeuroEngineering and Rehabilitation* 10, 19. doi:10.1186/1743-0003-10-19
